# A quasi-experimental mixed-method pilot study to check the efficacy of the “SOUND” active and passive music-based intervention on mental wellbeing and residual cognition of older people with dementia and dementia professionals’ burnout: a research protocol

**DOI:** 10.3389/fpsyg.2024.1327272

**Published:** 2024-02-14

**Authors:** Sara Santini, Alessandra Merizzi, Ioana Caciula, Maria Joao Azevedo, Albert Hera, Lena Napradean, Mirko Di Rosa, Sabrina Quattrini

**Affiliations:** ^1^Centre for Socio-Economic Research on Aging, IRCCS INRCA-National Institute of Health and Science on Aging, Ancona, Italy; ^2^Asociatia Habilitas – Centru de Resurse si Formare Profesionala, Bucharest, Romania; ^3^Associação-Sons do Estaminé, Trofa, Portugal; ^4^Associazione Centro Musicale Alessandro Orlandini-ACMO, Ancona, Italy; ^5^Scoala de Pian by Lena Napradean, Bucharest, Romania; ^6^Centre for Biostatistics and Applied Geriatric Clinical Epidemiology, IRCCS INRCA-National Institute of Health and Science on Ageing, Ancona, Italy

**Keywords:** music, dementia, non-pharmacological intervention, well-being, cognitive function

## Abstract

**Purpose:**

The SOUND method offers an innovative blended intervention based on music circle-activities and cognitive stimulation approaches which was co-designed by musicians, health professionals, older people with dementia, family caregivers and researchers, for its application in dementia settings. The purpose of the paper is to describe the detailed procedure of the quasi-experimental pilot study.

**Method:**

The experimental phase of SOUND uses a mixed-method design encompassing qualitative and quantitative observations, cognitive testing, self-report and interviewer-assisted questionnaires to investigate the effectiveness of the intervention for 45 people with dementia and 45 professionals (15 in every study country: Italy, Portugal, Romania).

**Results:**

The pilot study will be the first implementation of the SOUND intervention aiming to investigate the feasibility and preliminary effects of the method.

**Conclusion:**

The novelty of SOUND is its multicomponent method, including the most evidenced features for improving the wellbeing of participants.

## 1 Introduction

Dementia is an umbrella term to describe a set of cognitive, psychological and behavioral symptoms caused by brain diseases or conditions which are often progressive and non-reversible. The most common form of dementia, 60–70% of cases, is Alzheimer’s disease. Dementia is mostly prevalent in older age, with a significant increase over time as it almost doubles every 5 years after the age of 65 (World Health Organization, 2021). It is estimated that over 55 million of people live with dementia worldwide of which 9.780.678 in Europe (Alzheimer Europe, 2019; World Health Organization, 2021). This global epidemic is expected to almost triple by 2050 because of population ageing particularly in low and middle-income countries (World Health Organization, 2021).

There is no cure for dementia, although certain pharmacological treatments may slow down the progression of the disease. Nevertheless, evidence shows that pharmacological therapies for treating the psychological and behavioral symptoms of dementia (BPSD) have limited efficacy, severe adverse effects and increased mortality. Consequently, it is recommended to use non-pharmacological treatments (NPT) as a first-choice intervention (Magierski et al., 2020; Carrarini et al., 2021). In fact, NPT can be complementary treatments as, with a minimal risk for adverse effects, they can prevent and reduce BPSD, endorse quality of life, improve or maintain cognition and positively change brain activity (Dyer et al., 2017; Chalfont et al., 2018; Shigihara et al., 2020). A range of NPT are available, such as socio and psycho-educational approaches, cognitive and emotion interventions, physical exercise and sensorial activities (e.g., music, art and massage therapies). Other psychoeducational interventions are linked to training professionals and informal caregivers in order to reduce their stress/burnout and to improve their knowledge on dementia care (Barbosa et al., 2014; Briones-Peralta et al., 2020). Among all, music interventions targeted to older people with dementia seem to be the most effective NPT to manage BPSD (Abraha et al., 2017; Dyer et al., 2017). Indeed, music is associated to mental wellbeing, quality of life, self-awareness and coping in people with diagnosed health conditions and to reduced risk of depression in older people (Daykin et al., 2017). Moreover, music training looks like a powerful means for preventing the neurocognitive degeneration, since music enhances cerebral plasticity and induces the creation of new connections in the brain (Hyde et al., 2009; Habibi et al., 2017).

Musical leisure activities seem to have a positive effect on the cognitive, emotional, and neural function of older people both during normal aging (Klimova et al., 2017; Särkämö, 2018) and with dementia (Särkämö, 2018). Considering the latter, music can improve various aspects of their health and well-being: (a) cognition, especially verbal fluency and attention; (b) psychological aspects, such as mood, sense of self and identity; (c) mobility and coordination; (d) and behavior by reducing agitation, buffering isolation and strengthening communication (Brancatisano et al., 2019). Some studies show that music interventions can improve the cognitive state (Moreno-Morales et al., 2020), attention, immediate and delayed memory, executive function and gait speed (e.g., Domínguez-Chávez et al., 2019).

There is a great variety of music interventions including for instance, listening to music, playing instruments, and singing in chorus, but it is still not clear which is the most effective type of intervention and what are the factors determining their effectiveness. A relatively recent systematic review (Moreno-Morales et al., 2020) underlines that interventions involving listening to music have a greater positive effect on cognitive functions compared to active musical activities, such as singing or playing an instrument. In fact, listening to music implies a wide cortical activation by requiring the integration of perception of sounds, rhythms, and lyrics, alongside the simultaneous attention to the environment (Gaser and Schlaug, 2003; Soria-Urios et al., 2011; McDermott et al., 2012).

Many interventions, for example, are based on music therapy, i.e., an evidence-based practice carried out by trained and certified music therapists. Music therapy treatments can improve cognition (Bruer et al., 2007; Chu et al., 2013) and verbal fluency (Brotons and Koger, 2000; Lyu et al., 2018) whilst they can reduce associated symptoms of dementia such as depression (Chu et al., 2013) and agitation (Lin et al., 2010; Raglio et al., 2010; Vink et al., 2012; Tsoi et al., 2018). Music therapy can also have positive effects on clinical parameters by decreasing the systolic blood pressure of older people living in nursing home (Uğur et al., 2016).

Furthermore, group music-based treatments, i.e., based on passive and active music making exercises, not necessarily music therapy driven, can also reduce apathy (Tang et al., 2018), agitation (Choi et al., 2009; Ho et al., 2018) and depression (Ashida, 2000) and improve overall cognition (Cheung et al., 2016; Tang et al., 2018), verbal fluency and memory (Cheung et al., 2016). Thomas et al. (2017) demonstrated that individualized music treatments, such as personalized playlists, can have a positive effect on individuals’ mental health by reducing the use of antipsychotic medication with people with dementia.

Nevertheless, there is no agreement in the literature around the effectiveness of music-based intervention in different realms among older people with dementia, as research is still in initial stages. For example, Van der Steen et al. (2017) underlines that there is no evidence that music-based therapeutic interventions have effects on agitation or aggression, on emotional well-being or quality of life, nor on behavioral problems. Conversely, Moreno-Morales et al. (2020) conclude that there is evidence that music therapy can improve cognitive function in people living with dementia, that it can have a positive effect in the treatment of long-term depression and it can improve quality of life of people with dementia in the short-term.

The lack of evidence around the effectiveness of music interventions with OPDs, may depend on the studies small sample size, unclear assessment methods, the variety of music interventions (Van der Steen et al., 2017) and of tests used for the outcomes assessment (Moreno-Morales et al., 2020) and the absolute dearth of longitudinal studies that can demonstrate the long-term effect of music interventions on OPDs’ mood, cognitive and physical function (Moreno-Morales et al., 2020).

Non-pharmacological treatments, including music-based intervention, can have a positive effect also on healthcare workers (Cabrera et al., 2015). A qualitative study on music and dance interventions in dementia care found that healthcare staff had increased positive interactions, relationships, communication, sense of confidence, empathy, and an improved understanding of residents’ emotional state and experience of limitations (Melhuish et al., 2016). Such aspects, together with the team cohesion, work engagement and job satisfaction, contribute to preventing stress and burnout of healthcare staff (Maslach and Leiter, 1999; Öhman et al., 2017; Costello et al., 2018). However, there are few music-based treatments targeted to care professionals and none, to the best of our knowledge, involving both dementia care professionals and older patients.

Thus, even if the practice and part of the scientific literature, confirm the power of music in improving OPDs’ functions and care professionals’ well-being at the work place, the quality of evidence is low and so further research is needed (Dyer et al., 2017).

Considering the above, further studies should include emotional well-being and social outcomes to improve the knowledge about the effects of music on older people with dementia and dementia care professionals to support the implementation of music interventions as a care strategy.

This protocol has been designed to cover this knowledge gap by testing an intervention based on active and passive music activities delivered in circle with older people with mild cognitive impairment and mild to moderate dementia, and dementia care professionals. Particularly, the study aims to investigate two principal factors: 1) the efficacy of the SOUND intervention on OPDs; and 2) the impact of implementing the SOUND intervention on the DCPs who are delivering it as a novel group. Overall, the pilot study aims to assess the feasibility and preliminary outcomes of the protocol. The study is part of the SOUND project, funded by the Erasmus + program (contract 2021-1-IT02-KA220-ADU-000033494) and aimed at designing an original music-based curriculum for dementia care professionals and testing a pilot music-based intervention with OPDs in Italy, Portugal and Romania. At the time of writing this manuscript, we were about ending the training for dementia care professionals and planning the intervention that will be concluded in the three countries in early 2024.

## 2 Materials and methods

### 2.1 Study design

The pilot study will adopt a mixed-method approach encompassing quantitative and qualitative analysis of data gathered from video recording and from psychometric and idiosyncratic tools during specific times of the trial (pre-post and longitudinal). This design was chosen in preparation of a future full-scale study that will be a non-randomized larger trial. The latter seems to be in line with the features of the intervention and with the target population. In fact, the intervention is based on the SOUND methodology that can be carried out in groups including seven or eight OPDs coupled by the same number of care professionals, according to the guidelines for music-based interventions for people with dementia recommending the involvement of few persons in every group for ensuring the effectiveness of the intervention (Janus et al., 2020).

Moreover, being aware that quantitative measures alone would not be able to fully capture the possible effect of a non-pharmacological treatment on older people with a degenerative disease such as dementia, the study design also included the collection of qualitative variables e.g., through biographies and live and *ex post* observations. This very time-consuming methodology would not be feasible in a randomized controlled trial that would pre-suppose a very large sample of subjects.

In light of the above, this pilot study, as well as the full-scale study, will adopt a sampling for meaning procedure (Luborsky and Rubinstein, 1995): people will be selected who can provide knowledge and meanings useful for understanding of individuals’ experience by taking the insider’s perspective. The latter is meaningful and informative, because has, in itself, individuals’ key symbols, values and ideas that shape and inform their experience with dementia in the role of professionals and older people with dementia (Luborsky and Rubinstein, 1995). Accordingly, the sample size considered the six sampling-based considerations by Onwuegbuzie and Collins (2017) for sampling by meaning.

This pilot study will also assess the feasibility of data collection methods and intervention implementation (recruitment, retention, intervention fidelity, acceptability, adherence to the intervention, and engagement) not only for the larger scale study but also in the view of the application of the intervention with people with more severe dementia or in rehabilitative units (Teresi et al., 2022). The feasibility assessment will be carried out by a senior researcher in the role of supervisor who will analyze the data collection method and the intervention implementation according to the guidelines provided by Teresi et al. (2022).

**FIGURE 1:**
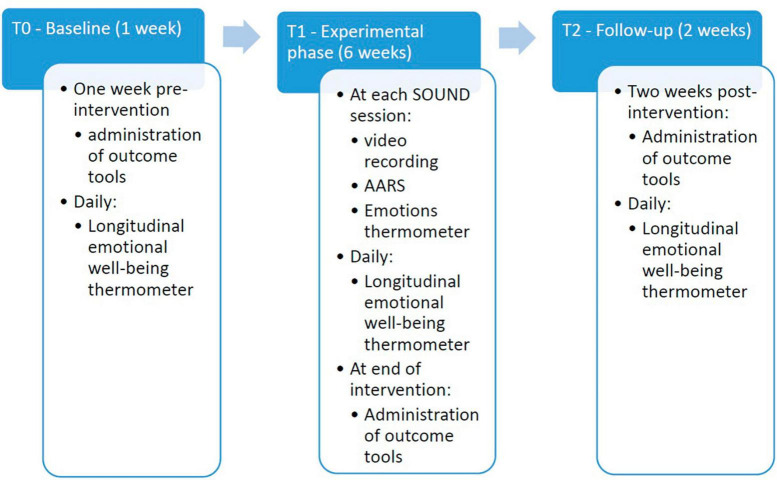
Research design.

The study includes dementia care professionals (DCPs) (e.g., neuropsychologists, physicians, educators, nurses, professional caregivers), and older people with dementia (OPDs) that will be monitored in different phases, depending on the type of variable and instrument used. Some data will be analyzed by a pre-post group comparison. The data will be collected in three moments: one week before the start of the intervention (T0), at its end (T1), and 2 weeks after the end of the intervention (T2). Data will be also collected during the intervention phase and analyzed longitudinally depending on the time frame of the collection (daily from T0 to T2 versus at each SOUND session). The Figure 1 below shows the progression of the data collection and experimental phase within the Research design.

### 2.2 Conditions

#### 2.2.1 Participants

In every study country, 15 OPDs and 15 DCPs will be recruited and participate to the experimentation, for a total of 45 OPDs and 45 DCPs (90 people overall). OPDs and DCPs’ inclusion and exclusion criteria are summarized below (Table 1).

**TABLE 1:** Participants’ selection criteria.

Type of participants	Inclusion criteria	Exclusion criteria
OPDs	• Interest in the project and signed consent form • Age 65 + • To be able to see, hear and move • Diagnosis of MCI or dementia with mild to moderate impairment (MoCA total score ≥ 10/30) • Absence of aphasia (mild acceptable) • Be able to understand and undertake simple task as required during activities	•Lack of consent to participate • Age 65− • Not be able to see, hear and move even with the help of appropriate aids • Absence of formal diagnosis of MCI or dementia or severe level of impairment (MoCA total score ≤ 10/30) • Moderate to severe aphasia • Not able to understand and undertake simple task as required during activities
DCPs	• Having experience in the dementia field (e.g., educators, occupational therapists, physiotherapists, psychologist, sociologists, nurses, music therapists, professional caregivers, doctors) • Having interest in the project and signed consent form • For the (co-)facilitators and internal/external observers: having completed the SOUND training	• Lack of experience working in the dementia field • Lack of consent to participate • For the (co-)facilitators and internal/external observers: lack of completion of the SOUND training

Older people with dementias will be recruited from the study organizations, health care dementia services and community services such as diagnostic centers, dementia day care centers, residential care facilities and charities. Individuals will already have a formal diagnosis of MCI or dementia. Their level of cognitive impairment is routinely assessed by the health professionals of the organization to which they belong. Therefore, these health professionals will support the recruitment and selection processes. Ethical approval has been obtained depending on the national legal requirements. OPDs retention will be empowered by collaborating with their professional caregivers who will present the SOUND activities as a pleasant novelty and as a gift.

DCPs will be selected among those who have successfully completed the SOUND training i.e., a 22-h training based on an original SOUND curriculum^1^ co-designed with OPDs, DCPs and family caregivers in Summer 2022. The training was implemented in Spring 2023 in the three study countries, by means of an e-learning platform, called Virtual Music Circle (VMC), specifically developed within the SOUND project, and of face-to face lessons, to train the professionals who will be involved in the intervention. The SOUND training concept and learning outcomes are deepened in a dedicated paper (Quattrini et al., under review)^2^. The training was open to entire teams of participating organizations. Selected DCPs will include permanent staff and trainees who may or may not have an established relationship with OPDs. DCPs will be required to study OPDs biographies to properly relate to OPDs during the intervention considering their needs and characteristics. None of the selected DCPs used the SOUND intervention prior to the trial, therefore all of them will apply it for the first time. The total number of people per every SOUND activities group will be of about 15, including both OPDs and DCPs. For example, one group may include eight OPDs, five DCPs, the facilitator and the co-facilitator. The other group may be made of seven OPDs, four DCPs, the facilitator and the co-facilitator. This will be made to guarantee the correct delivery of the intervention and provide the conditions to enhance its potentials. In fact, small-sized units of 5–15 people can positively impact older people’s well-being, behavior, functioning, and activity engagement (Verbeek et al., 2009) and doing activities in small groups can hinder the participants’ cognitive decline (Kok et al., 2016) and favor social connection (Van Zadelhoff et al., 2011) more than in large groups.

An attendance sheet will be used to monitor participation, whereby absence from half or more of the sessions will result in a drop-out.

#### 2.2.2 The SOUND intervention

The intervention will be implemented in Italy, in an Alzheimer day-care center, and in Portugal and Romania, in older people care facilities hosting people with dementia, where the DCPs who will be involved would regularly work. It includes 12 sessions delivered twice a week, for a 6-week period. Although there is no consensus regarding the frequency required for music-based interventions (Moreno-Morales et al., 2020), past research suggests that a higher occurrence may be more impactful (Carr et al., 2013).

During the intervention, OPDs and DCPs are positioned in a circle and everyone plays a specific role within the circle. OPDs will be the participants to whom the intervention is delivered. DCPs will implement the intervention by undertaking specific roles, i.e., the roles of facilitator, co-facilitator or internal observer. The role of facilitator will be assigned to a musician or a DCP with music skills, previously trained in the SOUND method. During the sessions, the facilitator proposes the activities to the participants in a responsorial style, holds the circle, welcomes and repeats any spontaneous and unexpected activity coming from the participants and proposes it to the whole circle. A DCP will be the co-facilitator, who is responsible for supporting the facilitator during the session. The role of internal observers, generally four per SOUND session, will be assigned to the rest of recruited DCPs: their task is to support the OPDs during the intervention in a non-intrusive and facilitating way while taking part in the activity. Facilitator, co-facilitator and internal observers take mental notes of their observations for reporting them in writing at the end of each session by using the live monitoring tools reported in Table 2. Four researchers (for covering all the sections of the circle circumference) will be the external observers: they will focus on OPDs’ emotional and behavioral responses to the proposed activities and take notes through dedicated tools (Table 2).

**TABLE 2:** Live monitoring outcomes tools.

Target group	Tools	Variable	Point of view	When it is used
Dementia care professionals	Video recording	Behavior	Video camera (researcher)	During SOUND sessions
	Live Session Emotions Thermometer	Emotional state, behavioral reaction	Self-report	After each SOUND session
Older people with dementia	Video recording	Behavior	Video camera	During SOUND sessions
	Live Session Emotions Thermometer	Emotional state, behavioral reaction	Internal observer	During SOUND session
	AARS	Affect	External observer	During SOUND sessions

Each SOUND session lasts about 45 min and it is divided in four different phases: (1) welcoming; (2) opening activity; (3) three to five main activities depending on the length and intensity of each one; (4) closing activity. To every phase, except for phase 3, an exercise corresponds.

The intervention foresees both active (vocal and rhythmic production) and passive (listening to pieces of music) music-based activities. Additionally, narrative activities may be linked to the music, such as creating or telling stories, talking about pictures, describing an object and so on. All activities have the general objective of enhancing participants’ wellbeing. Additionally, each activity aims to stimulate specific cognitive abilities. The goal is not to complete an exercise correctly, on the opposite the facilitator has the task to adapt activities to the group participants and use what is generally considered as a mistake as a resource to creatively change the activity. Therefore, the focus is on inclusion rather than on performance.

All the activities are chosen based on: (a) OPDs’ personal music preferences identified by means of a biographical sheet, as detailed below; (b) OPDs’ level of impairment; (c) the objective of the stimulation activities e.g., verbal fluency, memory, coordination. Activities are also organized taking into account particular fears and triggering factors of OPDs based on their personal stories and current situations, checked through the biographical sheet.

The activities proposed are personalized based on the OPDs’ preferences and on their actual mental and physical condition, thus they may need to be adapted from time to time and from person to person. For an example of activities, see Annex 1.

#### 2.2.3 The environment

The literature shows that there is a connection between certain characteristics of the environment and OPDs’ physical functioning, emotional well-being and social interaction (Calkins, 1988, 2001; Day and Calkins, 2002; Marcus, 2007; Marquardt and Schmieg, 2009; Chaudhury and Cooke, 2014). Chaudhury et al. (2016) underlines the need for creating physical environments appropriate and responsive to cognitive abilities and functioning of OPDs living in residential facilities and day care centers.

It is well-known that since neurological disorders can alter OPDs’ senses, i.e., their perception of reality, especially the sight, well-designed environments can promote wayfinding and orientation (Marquardt and Schmieg, 2009), improve activities of daily living function (Reimer et al., 2004), autonomy and meaningful activity (Kane et al., 2007), and reduce anxiety, agitation, aggression (Schwarz et al., 2004).

Homelike environments (i.e., open-plan lounge/dining areas, residential furniture and flooring) displayed reduced verbal and overall aggression, verbal agitation and anxiety (Zeisel et al., 2003; Wilkes et al., 2005), are associated with fewer walking/pacing episodes, (Yao and Algase, 2006) and enhance engagement in daily activities, social interaction (Campo and Chaudhury, 2011) and autonomy (Chaudhury et al., 2016).

High noise levels, such as for example alarms, rings and staff conversations not including older users, are associated with the latter’s reduced social interaction, increased agitation and aggression, disruptive behavior and wandering (Campo and Chaudhury, 2011; Garcia et al., 2012; Garre-Olmo et al., 2012; Joosse, 2012).

The intensity of the light is also important for creating a welcoming environment for OPDs. In fact, the exposure to a bright light can decrease agitation and disruptive behavior and improve cognition and mood, albeit modestly (Nowak and Davis, 2010).

This is the reason why it is very important that the room hosting the SOUND activities is homelike displayed, welcoming, with soft light and that the level of noise is very low, for favoring concentration and cognition. The room will need to be large enough to allow participants movement, tidy with as little distracting objects as possible, with a suitable level of light which is not too dark and not too bright, and with small sound reverberation. The chairs need to be arranged in a circle with assigned seats (placing a sheet on each chair, where the name of each participant is written) taking into account specific aspects for interacting with the OPDs: (a) interpersonal dynamics; (b) visual/auditory difficulties; (c) need for proximity to the healthcare staff; (d) definition of roles. Considering the space of the circle as inside a square, one chair for each corner needs to be positioned outside the circle for the external observers.

### 2.3 Outcome measures and data collection tools

#### 2.3.1 Outcome measures

The primary outcome variable for OPDs is the level of their mental wellbeing, while the primary outcome variable for care professionals is the level of burnout. Secondary outcomes variables for DCPs are (a) level of stress, (b) level of work cooperation and of (c) emotional well-being and, for OPDs, they are levels of (a) cognitive abilities, (b) neuropsychiatric symptoms, (c) mood and (d) emotional well-being.

Pre, post and longitudinal-intervention outcome (Table 3) as well as live monitoring tools (Table 2) were developed for assessing the impact of the SOUND intervention on DCPs and OPDs.

**TABLE 3:** Pre, post and longitudinal-intervention outcome tools.

Target group	Tools	Variables	Who administers the tool	Who answers the questions	When it is used
DCPs	Burnout Assessment Tool (BAT)	Stress	Care professional	Care Professional	T0, T1, T2
	*Ad hoc* questionnaire	Stress	Care professional	Care professional	T0, T1, T2
	*Ad hoc* questionnaire	Work cooperation	Care professional	Care professional	T0, T1, T2
	Longitudinal Emotional Well-being thermometer (LEWT)	Emotional wellbeing	Care professional	Care professional	Daily report from T0 to T2
OPDs	Neuropsychiatric Inventory (NPI)	Neuropsychiatric symptoms (patient) and Caregiver Distress	Psychologist	Family caregiver	T0, T1, T2
	MoCA	General cognition	Psychologist	Older person	T0, T1, T2
	FAB	Cognition – Executive functions	Psychologist	Older person	T0, T1, T2
	HADS	Mood	Psychologist	Older person	T0, T1, T2
	WHO (Five) Well-Being Index (WHO-5)	Wellbeing	Psychologist	Older person	T0, T1, T2
	Longitudinal Emotional Well-being Thermometer (LEWT)	Emotional wellbeing (of patient)	Family caregiver	Family caregiver	Daily report from T0 to T2

#### 2.3.2 Pre-post and longitudinal tools

Concerning DCPs, an *ad hoc* questionnaire has been developed to investigate the level of stress and cooperation of professionals before and after the intervention. Beyond the demographic questions (i.e., age, gender, nationality, marital status, education), the pre-intervention questionnaire includes *ad hoc* questions on: (a) work condition (e.g., position, role, activities); (b) music attitudes; (c) well-being, motivation, satisfaction and self-realization at work (d) teamwork, (e) relationship with patients and family caregivers. Additionally, the Burnout Assessment Tool (BAT) will be administered for evaluating the work-related stress. Respondents can answer all the questions (i.e., both *ad hoc* questions and the BAT battery) through a five-point Likert scale, where 1 represents the lower and 5 the higher score/frequency. The post-intervention questionnaire for DCPs follows the same structure of the pre-intervention ones, except for quantitative and qualitative questions asking to what extent and how the intervention has improved every area e.g., well-being, team work, relationship with OPDs.

The Burnout Assessment Tool (BAT) is a self-assessment questionnaire that measures parameters associated with burnout (De Beer et al., 2020; Schaufeli et al., 2020) validated in Italy (Angelini et al., 2021; Borrelli et al., 2022), in Romania (Oprea et al., 2021), in Portugal (Sinval et al., 2022) and with European cut-off scores (Schaufeli et al., 2023). The BAT contains four different subscales: exhaustion, mental distance, loss of emotional control and loss of cognitive control. In addition, there are two sub-dimensions: psychological disorders and psychosomatic disorders. In total, the questionnaire consists of 33 items (23 for the reduced version), each with a 5-point scale (1 = never, 2 = rarely, 3 = sometimes, 4 = often and 5 = always). The total score is obtained by summing the points. A difference of ± 10 points with a 95% confidence interval shows significance in the variation of burnout (Daniels et al., 2022). The BAT will be the tool to assess the primary outcome for DCPs.

For the OPDs, demographic and baseline data will include age, gender, nationality, marital status, education, living condition, dementia service they attend, type of diagnosis, time of diagnosis, level of verbal expression and comprehension. As outcome tools, the following psychometric and standardized measures will be used.

The Neuropsychiatric Inventory (NPI) is a tool to assess dementia-related behavioral symptoms by examining 12 sub-domains of behavioral issues: delusions, hallucinations, agitation/aggression, dysphoria, anxiety, euphoria, apathy, disinhibition, irritability/lability, aberrant motor activity, night-time behavioral disturbances and appetite and eating abnormalities (Cummings et al., 1994; Cummings, 1997, 2020). The inventory is administered by a health professional to the primary family (if the older person lives in the community) or professional caregivers (if the older person lives in a care home). Each sub-domain includes a screening question followed by a sub-list of questions to answer if the behavior is present, which rate the frequency (4-point scale) and severity (3-point scale) of symptoms plus the level of distress caused to the caregiver (5-point scale). The measure provides a total score for BPSD ranging 0–144 and for caregiver’s stress from 0 to 60. The validity and reliability of the tool is well established even across translations (Farina et al., 2009; Ferreira et al., 2015).

The Montreal Cognitive Assessment (MoCA) is a brief screening tool for detecting mild cognitive impairment and dementia, which is divided in seven domains and sub-scores: orientation (6 points); attention (6 points); memory (5 points for delayed recall); visuospatial/executive (5 points); naming (3 points); language (3 points); abstraction (2 points); if the person has ≤ 12 years of education a further point will be added (Nasreddine et al., 2005). The test has been shown to have good validity, internal consistency, test–retest and inter-observer reliability (e.g., Freitas et al., 2013) and it is used worldwide.

The Frontal Assessment Battery (FAB) is a short neuropsychological tool aiming to assess executive functions or functions related to the frontal lobes and correlated with frontal metabolism (Sarazin et al., 1998). It includes 6 subtests one for each investigated cognitive function: similarities test (conceptualization and abstract reasoning); verbal fluency test (mental flexibility); Luria motor sequences (motor programming and executive control of action); conflicting instructions (sensitivity to interference); go–no go test (inhibitory control); prehension behavior (environmental autonomy). The scoring for each subtest is from 0 to 3, for a maximum total score of 18 (highest performance). The battery has good validity (correlation of ρ = 0.82 with the Mattis Dementia Rating Scale) and interrater reliability (κ = 0.87) (Dubois et al., 2000).

The Hospital Anxiety and Depression Scale (HADS) is a 14-item self-report measure for screening anxiety (7 questions) and depression (7 questions) states. The Likert-scale ranges from 0–3 for a maximum total score of 21, with higher scores indicating greater anxiety and depression, and with a cut-off > 10 (Zigmond and Snaith, 1983). It has shown high concurrent validity with other widespread anxiety and depression assessments and has proved a 20-day test-retest reliability of0.94 (Michopoulos et al., 2008). It is widely used in the field of dementia as it is brief, measures both anxiety and depression and is suitable for people with physical comorbidities (e.g., Clare et al., 2012).

The WHO Wellbeing Index (WHO-5) is a self-assessment tool to measure the subjective mental well-being of individuals which is validated for older people (Heun et al., 1999). It includes five items each scored from 0 to 5. The total score ranges from 0 to 25, with higher scores indicating greater mental well-being. Scores < 13 indicate poor well-being and suggest depression according to the criteria of the International Statistical Classification (Tiganov et al., 1997). It is recommended to transform the raw scores to percentage scores, by multiplying it by 4, for data analysis when investigating change over time (Topp et al., 2015). Low mood may be suggested by a percentage score of ≤ 50 whilst a score of ≤ 28 may indicates depression. Similar to Kikuchi et al. (2023), a variation of ± 10 points of the percentage score with 95% confidence intervals will be considered significant. The reliability and validity of the WHO-5 is well established (e.g., Bech, 2012; Takai et al., 2013). The WHO-5 will be the tool to assess the primary outcome for OPDs.

The SOUND consortium developed the Longitudinal Emotional Well-being Thermometer (LEWT), an idiosyncratic tool for monitoring the emotional well-being of DCPs and OPDs from T0 to T2 on a daily basis for the overall duration of the intervention. This is a self-report tool for the professionals and an observation-based tool for primary family or professional caregivers to record OPDs’ emotional state. Both versions include the picture of a colored thermometer, with each color indicating an emotional range and corresponding to a score. The color and the scoring have been assigned on the basis of the quality and level of neurophysiological arousal (i.e., parasympathetic and sympathetic systems): green indicates a homeostatic state of peace and calm (score = 0); darker green is joy/satisfaction whilst yellow indicates confidence/hope (score = 1); light blue means worried/anxious versus orange is annoyed/frustrated (score = 2); dark blue indicates sad/depressed whilst red means angry/disgusted (score = 3). The higher the score the greater the emotional distress. DCPs will be asked to rate their prevalent emotional wellbeing state every day, at the end of the day, for 63 days. Likewise, primary family or professional caregivers, who have a pre-existing relationship with the OPD and are able to observe them daily, will be asked to report their cared for mood. The following mean range can be obtained from the total sum divided by the total days: scores from 0 to 1 indicate well-being, scores from 1 to 2 indicate mild emotional discomfort, and scores from 2 to 3 indicate emotional distress. Each emotional state will also have a code 1–7 to identify the type of emotion and calculate its prevalence throughout the intervention. Additionally, both versions include a final column in order to: (a) rate (on a 1–5 scale ranging from “not at all” to “completely”) how much the recorded emotion may be linked to SOUND sessions for health professionals; (b) record meaningful events in the daily life of OPDs which may be related to their emotional state. The results from the scoring can be summarized in a graph showing the daily trend of the emotional arousal, the frequency of each emotional group and their relationship to the SOUND intervention. The minimum detectable change and the threshold of clinical improvement will be calculated.

#### 2.3.3 Live monitoring tools

The live monitoring of the observed OPDs’ emotional and behavioral reactions is very important because the deficit in the short-term memory of OPDs does not allow to gather reliable data about their emotions and thoughts, even if asked at the end of the intervention session. Thus, the use of further tools for monitoring the effects of the intervention during the delivery of the SOUND activities is included (Table 2).

DCPs and OPDs, who will have provided their written or audio recorded verbal consent, will be video recorded by two cameras (for having two points of view and minimize blind points) during the delivery of the SOUND sessions. The recordings will be watched by the researchers who will analyze and interpret participants’ behaviors with the aim to evaluate the method and improve it.

Collecting information on OPDs and DCPs’ emotions is important to understand if there is an emotional synchronization between the two types of beneficiaries, that may contribute to overcome the professional-patient asymmetry and so increase the chances for a successful intervention. Such observations can be collected from the analysis of the video recordings. Furthermore, the research protocol includes an *ad hoc* instrument for this purpose, the Live Session Emotions Thermometer (LSET)^3^. LSET is an idiosyncratic tool for monitoring the emotions of both DCPs and OPDs during the activities which includes two forms, one for recording the OPDs observed emotional reaction and one for noting the professionals’ own reaction and how they handled the situation. This form encompasses quantitative and qualitative data. The tool includes the image of a thermometer with 10 degrees, where 1 indicates the lowest and 10 the highest intensity of the emotion. In the first form, the observer (DCP) identifies and writes down the prevalent emotion of the observed person (OPD) e.g., happiness or sadness, scores its intensity by crossing the corresponding degree in the thermometer, and describes the observed behavioral reaction and the activity that took place in that moment. In the second form, DCPs will report their own emotion, scored by intensity, their thoughts, their behavioral reaction (strategy adopted to handle the situation) and their sense of efficacy regarding the occurred episode. DCPs are requested to fill the LSET immediately after every SOUND session.

In addition, the external observers (i.e., the researchers), will be focused on each OPD and will fill-in the Apparent Affect Rating Scale (AARS; Lawton et al., 1999). The AARS is an observational tool designed for research purposes in the dementia field. The scale aims to rate five emotions, two of which positive (pleasure and interest) and three negative (fear/anxiety, anger and sadness), and their duration on a scale from 0 to 5 (0 = can’t tell; 1 = never; 2 = less than 16 secs; 3 = 16–59 secs; 4 = 1–2 mins; 5 = more than 2 mins) by observing the person for 5 min.

#### 2.4 Data analysis

The analysis plan includes, for quantitative data, outcomes description. Normality in distribution of continuous variables will be assessed via Shapiro-Wilk test and the following measures will be reported: mean and standard deviation for normally distributed variables or median and interquartile range for non-normally distributed variables. Absolute frequency and percentage will be reported for categorical variables. Comparisons between outcomes and exposures will be made using the Chi Square test, (in the case of categorical variables) or *t*-test or F-Anova (in the case of comparisons between normally distributed continuous variables and the groups), or by non-parametric tests such as Wilcoxon rank-sum test or Kruskal-Wallis test (in the case of comparisons between non-normally distributed continuous variables and the groups). Through Pearson’s or Spearman’s correlation we are going to study the relationship between continuous variables as appropriate according to variables’ distribution. Temporal comparisons (T0 vs. T1, T0 vs. T2, or T1 vs. T2) will be conducted by *T*-test for paired samples. Possible multivariate models will be assumed in case of significant differences in outcomes at the univariate level: coefficients and standard errors or odds ratios and 95% confidence intervals will be reported according to the typology of outcomes (continuous or binary). Goodness of fit will be determined by R-squared of pseudo R-squared as appropriate. Subjects withdrawing from the study will not be replaced, according to the intention to treat (ITT) principle. Sequential imputation using chained equations method will be applied in case of missing values in covariates. Statistical analyses will be conducted by a statistician, who will be blind to group allocation prior to analysis. The significance threshold will always be set at p < 0.05. The software used for the analyses will be SPSS for Win V24.0 (SPSS Inc., Chicago, IL, USA).

Qualitative data from the open-ended questions, included in the *ad hoc* questionnaire for professionals, in the LEWT and in the LEST, will be analyzed thematically (Braun and Clarke, 2006). The textual data will be analyzed by two independent researchers and checked by a third one (add reference). The notes taken by researchers during the watching of the SOUND sessions’ video recordings will be reported into a narrative (Kutsche, 1998) and all narratives will be summarized cross-nationally. Then the narratives will be coded to select and emphasize relevant information answering the research questions and the codes will be merged in themes (De Munck and Sobo, 1998). The textual data will be analyzed by two independent researchers and checked by a third one for minimizing the bias of subjectivity (Golafshani, 2003).

The datasets that will be generated and/or analyzed during the pilot study will be available from the corresponding author on reasonable request.

#### 2.5 Ethics

This protocol and the template informed consent forms have been reviewed and approved by the responsible local Institutional Ethical Committees, as required in each participating country with respect to scientific content and compliance with applicable research and human subjects’ regulations. Any modifications to the protocol which may impact on the conduct of the study, potential benefit of the patient or may affect patient safety, including changes of study objectives, study design, patient population, sample sizes, study procedures, or significant administrative aspects will require a formal amendment to the protocol. Any amendment to the protocol will be approved by the Ethics Committees.

Each type of subject involved will be duly and comprehensively informed about the objectives of the study and the modalities of the music-type intervention, through an information sheet but also through an interview with the scientific supervisor (Dr. SS) when requested. Older people with dementia will be provided with the informed consent and will be asked to sign it in the presence of the family caregiver and the same sheet will be given to the latter. In the information sheet, as well as during the interview, it will be emphasized that adherence to the study is completely voluntary and that it can be quit at any time without having to give any explanation. The type of intervention will be explained to both the person with mild-to-moderate dementia and their family caregivers according to a capacity approach and with full respect for the older person’s residual capacities. For the same principle, where possible, informed consent will be signed by the person with dementia and their family caregiver/legal guardian. The intervention does not anticipate any risk, discomfort or intrusion of privacy. The professionals involved will be registered healthcare practitioners with extensive experience in working with OPDs or musicians trained in dementia care and have the skills to identify and address any discomfort experienced by participants during the intervention. The participation will not require the suspension of concomitant care or interventions.

All study-related information will be stored securely at the study site. All participant information will be stored in locked file cabinets in areas with limited access. All reports, data collection, process, and administrative forms will be identified by a coded ID [identification] number only to maintain participant confidentiality. All records that contain names or other personal identifiers, such as informed consent forms, will be stored separately from study records identified by code number. All local databases will be secured with password-protected access systems. Forms, lists, logbooks, appointment books, and any other listings that link participant ID numbers to other identifying information will be stored in a separate, locked file in an area with limited access.

The scientific integrity of the study requires that the data from all SOUND sites (i.e., Italy, Portugal and Romania) be analyzed study-wide and reported as such. All results coming from the data collected through this protocol are expected to protect the integrity of the major objective of the study at the time of their dissemination through scientific papers and oral presentations, that will be agreed in the Steering Committee (made of the Authors).

The primary outcome papers of SOUND i.e., those reporting the effects of the intervention on OPDs’ wellbeing and DCPs’ burnout will be presented by the first author to the Steering Committee for approval as well as secondary outcome papers and presentations (e.g., on DCPs’ cooperation or OPDs’ reduction of neuropsychiatric symptoms). The study results will be released to the participating DCPs, OPDs, informal caregivers and the general medical community.

The activities and music are carefully chosen in order to elicit in participants only feelings of peace and well-being, as previous similar experiments have shown (Caldini et al., 2019). In compliance with the Declaration of Helsinki (stating that “the protocol should describe arrangements for post-study access by study participants to interventions identified as beneficial in the study or access to other appropriate care or benefits”), should this study provide evidence of the effectiveness of SOUND, the SOUND activities will be included in the routine of the dementia care centers. This goal is probable and achievable because a total of 63 professionals have been trained on the SOUND method, 29 in Italy, 17 in Portugal and 17 in Romania; moreover, the Virtual Music Circle training platform is predisposed to train further professionals (Quattrini et al., under review, see text footnote 2).

## 3 Discussion

To the best of our knowledge, SOUND is one of the few studies focusing on the impact of a music-based intervention on both dementia care professionals and older people with dementia, since the available studies are targeted to older people mainly (e.g., Hyde et al., 2009; Daykin et al., 2017; Habibi et al., 2017; Brancatisano et al., 2019; Domínguez-Chávez et al., 2019). Conversely, in this study, the former and the latter, are conceived by the protocol as distinct but also intertwined targets. They build together a unique care ecosystem that can be well-represented and interpreted by the circle, that is a democratic setting able to overcome the care asymmetry between care professionals and patients.

The intervention is innovative, because it is delivered in circle, it is led by facilitators with the support of other care professionals, all trained on the method through an original curriculum, and it includes different types of music activities both passive and active, highly personalized as based on the beneficiaries’ preferences and attitudes collected before the intervention finalization.

The protocol has been designed based on the recommendations of Moreno-Morales et al. (2020) by including well-focused outcome measures and discussing how the findings may improve the well-being of OPDs and DCPs.

Since the effects of music are not immediate (Moreno-Morales et al., 2020), SOUND was conceived as a progressive and continuous intervention to obtain successful results (Leubner and Hinterberger, 2017) and it foresaw both pre-post evaluation and medium-term follow-up.

In light of the above, with regard to OPDs we expect: (a) an increase of ± 10 points with 95% confidence interval in the WHO-5 percentage scores regarding wellbeing; (b) no decrease in MoCa and FAB scores regarding cognition; (c) a 50% reduction of the HADS score or absence of depression and anxiety concerning mood; (d) a decrease of 2 points corresponding to the median value for the BPSD symptoms and caregivers distress in the NPI; (e) a negative variation, e.g., of 0.5–1 points toward 0, in the LEWT as an index of improved emotional wellbeing.

Concerning the impact of the intervention on DCPs, we expect that: (a) the BAT scores will decrease by ± 10 points with a 95% confidence interval; (b) well-being at work, teamwork cooperation and communication with the OPDs will improve of one point on the five-point Likert scale for at least half of the items in each area. For example, to the question “How do you rate the quality of your relationship with older people with dementia?” we expect a minimal change from “Fair” to “Quite good”; (c) the LEWT scores will show a trend toward 0.

It is methodologically worth mentioning that this protocol was designed for DCPs daily working in a dementia care facility. However, the methodology may be applied even by external professionals who have an external collaboration with service providers. In this case, the impact of the caregiver stress, that may be influenced by working in a new workplace, could be measured by adding a question such as “In what way the delivery of the SOUND method in an unusual workplace have influenced your levels of work-related stress?”

One strength of the protocol lies in the use of both quantitative and qualitative data collection tools, and in a multi-method approach that will allow to gather written and visual data that will generate numerical, textual and narrative results. Another strength is the cross-national nature of the study that can provide interesting culture-based tips, since every team will use popular and traditional songs belonging to their context.

One limitation of the study may concern the small sample size at national level that will not allow a generalization of the results. Moreover, the protocol does not foresee the monitoring of clinical parameters such as cortisol, blood pressure as recommended by Uğur et al. (2016). In fact, although it would have been interesting to check the impact of the intervention on these realms, the study did not receive enough funding to cover the expenses for a biochemical investigation.

Another limitation of the protocol, may be the missing inclusion of family caregivers as a target of the study. In fact, it would have been interesting to investigate the effect of the intervention on the dyad relationship family caregiver-cared for e.g., on communication and empathy. The SOUND project is currently training a group of family caregivers for adapting music activities to the home context, through mini animated videos. However, the effectiveness of this approach will need a separate evaluation.

Additionally, the OPDs-DCPs relationship was not included as a variable to investigate. The protocol could be amended for a further study to investigate primarily the effects of the intervention on the quality of the patient-caregiver relationship.

Finally, methodologically, the data on OPDs collected through the observation of DCPs as internal observers and researchers as external observers may entail the risk of subjectivity. Observation may in fact, be influenced by factors like mood, the personal experience (e.g., having a relative with dementia), the desire that the intervention has an impact on participants and so on. Therefore, to ensure the study confirmability (objectivity) and also its credibility, transferability, dependability according to Lincoln and Guba (1985), Shenton (2004) and Golafshani (2003), several expedients should be adopted by the research team e.g., (a) the admission of personal predispositions by the observers and the awareness that they can influence their data perception and interpretation; (b) the use of specific tools that try to translate qualitative data in categories and quantitative data/scores e.g., the AARS and the LSET; (c) the triangulation i.e., the presence of more than one researcher observing the same person, and the analysis of the recorded videos of the sessions helping making a synthesis of different points of observation; (d) frequent debriefing sessions in the team; (e) the examination of previous research findings referring to the same issue; (f) the description of the research design and its realization, and details of data collection and analysis processes.

The main study challenge may lie on the need of a multidisciplinary team, made of researchers, dementia care professionals and trained musicians for properly applying the intervention and monitoring its impact on the target groups. Since the presence of researchers and musicians is not common in the older people care facilities, this may limit the replicability of the study. Conversely, the SOUND curriculum is available online as an open-access resource, through the Virtual Music Circle e-learning platform and it will be open access at the end of the project for any care professional or musician who wanted to learn the method and deliver the activities with OPDs.

Finally, the pilot study can provide useful insights for future studies and contribute to improve the knowledge about the effectiveness of the music in dementia care facilities for both professionals and older patients.

## Ethics statement

The study was approved by the IRCCS INRCA Ethic Committee on 26th May 2023 with General Director Communication number 234 provided on 9th June 2023.

## Author contributions

SS: Conceptualization, Data curation, Formal analysis, Funding acquisition, Investigation, Methodology, Project administration, Resources, Software, Supervision, Validation, Visualization, Writing−original draft, Writing−review and editing. AM: Conceptualization, Data curation, Investigation, Methodology, Writing−original draft, Writing−review and editing. IC: Conceptualization, Data curation, Investigation, Methodology, Writing−review and editing. MA: Conceptualization, Data curation, Investigation, Methodology, Writing−review and editing. AH: Conceptualization, Methodology, Supervision, Validation, Writing−review and editing. LN: Data curation, Investigation, Writing−review and editing. MD: Formal analysis, Software, Writing−review and editing. SQ: Conceptualization, Data curation, Investigation, Methodology, Project administration, Writing−review and editing.

## Footnotes

1
https://soundeuproject.eu/results/

2 Quattrini, S., Merizzi, A., Caciula, I., Napradean, L., João Azevedo, M., Costa, S., et al. (under review). *The design and implementation of a novel music-based curriculum for dementia care professionals. The experience of SOUND in Italy, Portugal and Romania*.

3 The Live Session Emotions Thermometer (LSET) was developed by Giorgia Caldini et al. during the first experimentation of Circleactivities at the day-care center Civica di Trento in 2018 (Caldini et al., 2019).
