# Supplementary material for: A quasi-experimental mixed-method pilot study to check the efficacy of the “SOUND” active and passive music-based intervention on mental wellbeing and residual cognition of older people with dementia and dementia professionals’ burnout: a research protocol
Source: Front Psychol. 2024 Feb 14;15:1327272. doi: 10.3389/fpsyg.2024.1327272 (PMC10901113; doi:10.3389/fpsyg.2024.1327272)
Supplement: Supplementary file 1 [file Table_1.DOCX]

**Annex 1**-**SOUND activities: objectives, materials, procedure and timing**

The table reports the details of activities for a single SOUND circle that have been tested in June 2022 during the co-design phase in Italy involving OPDs, DCPs and family caregivers (i.e. daughters, sons and spouses of OPDs). Such activities among others will underpin the development of the intervention in the three participating countries.

| Activity title | Main objective | Materials | Activity procedure | Timing |
| --- | --- | --- | --- | --- |
| Names | Ice-breaking | None | The facilitator welcomes (and proposes) to say your name starting from his/her and continuing counterclockwise. Then he/she invites them to stand up and sing or say their name in the way that everyone prefers and invites the circle to repeat the name of each person. The facilitator begins, and the others follow counterclockwise. | 5-10 min |
| Sticks | Stimulating the listening capability, attentiveness and coordination | Sticks | The facilitator, in an upright position like the rest of the circle, hands the sticks to the participants and illustrates the activity: facilitator will propose making sounds using the instruments and the participants will have to try to reproduce them. | 5 min |
| Coloured foulard | Stimulating coordination, body movement, sight, hearing and attention. | Foulards | The facilitator gives each participant a coloured foulard, trying to arouse general curiosity, then, sitting down, he/she introduces the "game", asking the participants to hold the scarf with their right hand, and to raise it high up, either when the facilitator calls its colour, or when he/she asks to rise it, no matter its colour. | 5 min |
| Story telling | Stimulating attention, memory and language fluency | None | The facilitator remains seated in the circle and introduces the activity, starting to tell that in the morning he/she saw, for example, something flying. Then the facilitator asks the participants questions to build a story together: each older person adds a detail, for example what is flying, and the participants agree that it is a kite, and then the colour of the kite, who makes it fly, where does it go, what is it possible to see from the kite’s position, etc. | 10 min |
| Body percussion | Stimulating the body coordination, the memorization of the proposed movements | None | The facilitator proposes a rhythm by clapping the hands/feet and invites users to replay. | 5 min |
| Listening to a popular song | Stimulating the body coordination |  | The whole group is sitting down listening in silence, when the facilitator gets his/her bag and tries to look for something in the bag. He/she finds a song, the group sings and follows the music. The facilitator encourages the group to move by saying “let’s move a bit” and by increasing his/her own movements. | 5 min |
| Images of the city | Stimulating memory and verbal fluency | Cards, pictures, images | The facilitator stands up and goes around the circle showing some pictures of the city and asking each individual to choose an image specifying that everyone can look at them and get the one that they like most.  At this point, a game of imagination and remembrance starts, where every participant recalls personal or public events that occurred in the places represented in the photographs.  The remembrances call for a song that the co-facilitator plays and the facilitator invites participants to move on the music. | 10 min |
| Coloured cloth | Stimulating memory and body coordination |  | The facilitator shows a cloth coloured with different colours in triangular segments with a vertex in the centre of the cloth. They then get the cloth out and the facilitator asks each participant to grasp a flap. The co-facilitator plays an instrumental, descriptive, evocative piece of music (e.g. The four Seasons of Antonio Vivaldi) and the facilitator remarks on the beauty of the cloth and begins to encourage an up and down movement accompanied by vocalisation “oooooh”. Everyone participates in having fun and the facilitator asks everyone if they like the colour of the clothes each one has in front of him/her and what does it bring to their minds. | 5 min |
| Coloured balloons | Many coloured balloons |  | The facilitator invites everyone to get a balloon and starts giving instructions, turned to the co-facilitator (or to an internal observer) who is the first to do so, followed by all the others who take a balloon or are helped to do so by the facilitator. The facilitator calls a colour and the persons who have the balloon of that colour raise it. | 5 min |
